# Adopting assistive technologies for the support and care of people with mild dementia living at home—Understanding the decision-making process: a qualitative study

**DOI:** 10.1186/s12877-025-05945-2

**Published:** 2026-02-05

**Authors:** Sarah Palmdorf, Tristan Gruschka, Gerrit Eliaß, Angela Nikelski, Eva Trompetter, Lea Stark, Christoph Karlheim, Christoph Dockweiler, Stefan H. Kreisel

**Affiliations:** 1 Institute for Educational and Health-Care Research in the Health Sector, Hochschule Bielefeld– University of Applied Sciences and Arts, Interaktion 1, Bielefeld, 33619 Germany; 2 Nursing Science Staff Unit, Franziskus-Hospital Harderberg, Alte Rothenfelder Str. 23, Georgsmarienhütte, 49124 Germany; 3 Faculty of Social Sciences, Hochschule Bielefeld– University of Applied Sciences and Arts, Interaktion 1, 33619 Bielefeld, Germany; 4 DGB NRW Ostwestfalen-Lippe, Marktstr. 8, 33602 Bielefeld, Germany; 5 Department of Psychiatry and Psychotherapy, Division of Geriatric Psychiatry, Evangelisches Klinikum Bethel, Universitätsklinikum OWL der Universität Bielefeld, Campus Bielefeld-Bethel, Bethesdaweg 12, 33617 Bielefeld, Germany; 6 Department Digital Biomedicine and Health Sciences, School of Life Sciences, Digital Public Health, Universität Siegen, 57076 Siegen, Germany; 7 Department of Innovation & Research, Evangelisches Klinikum Bethel, Universitätsklinikum OWL der Universität Bielefeld, Campus Bielefeld- Bethel, 33617 Bielefeld, Germany

**Keywords:** Assistive technologies, Assisted living, Qualitative methods, Caregiving, Person-centred care, Dementia

## Abstract

**Background:**

Assistive technologies may play a crucial role in addressing needs of people with dementia. While technical feasibility often drives development, the decision-making process that might lead to their adoption (or rejection) is not fully understood. We aim to explore contextual factors influencing this process and “necessities” technology should fulfil from the user-perspective, so that the use of assistive technologies can be better targeted– potentially fostering a more supportive home environment.

**Methods:**

In this qualitative study, interviews (8 people with dementia and 7 partners) and focus groups (7 focus groups included a total of 29 participants) with individuals with milder stages of dementia living at home and their support/care-network were carried out. Grounded Theory and Structuring Content Analysis were used to analyse the data.

**Results:**

Six main thematic categories outline the decision-making process. The “assessment of (one’s own) resources”, while evaluating the “(potential) benefits of assistive technologies” contrasts with “(potential) adverse effects” of their use (or their non-use respectively), shaping the context of the decision-making process. There is an appraisal of necessary “(pre-existing) trust” in technology. An a priori “openness towards assistive technologies” intersects latter aspects. A very substantial “need for information” is noted.

**Conclusions:**

While technical feasibility and tailored solutions are important, they are not the sole determinants of assistive technology adoption in this group. The desire to preserve self-determination and independence emerges as a key motive for choosing assistive technologies; technology can also be seen to invoke stress and negative emotions, and will consequently be rejected. Technology should, moreover, be perceived to be “meaningful” on different levels. Considering these points when developing technologies and addressing them when counselling those affected by dementia and their networks may “tip the scale” towards acceptance.

**Supplementary Information:**

The online version contains supplementary material available at 10.1186/s12877-025-05945-2.

## Background

Individuals with dementia and their support and/or care-network (e.g. partners, other relatives) often require assistance to maintain home care arrangements [1]. They carefully evaluate interventions based on their specific care needs, choosing the most suitable options [2, 3]. Assistive technologies can be one such support option [4, 5]; they are technical devices or appliances designed to enhance functioning, independence, and overall well-being [6]. There is some indication that once adopted, they have the potential to positively impact the quality of life, reduce burden, improve care, and foster social interaction– and enable those affected to stay in their own home environment [7]; recent evidence, however, questions these appraisals [8].

A review of research on requirements for people with dementia and their caregivers identified several important domains [9]: Assistive technology developers should prioritize solutions that are considered useful in addressing specific (care) needs, including support for daily activities, physical and/or psychological assessment, monitoring and security features; assistive technologies should meet technical and practical prerequisites such as user-friendliness and integration into existing support and care provisions; additionally, accessible information and guidance on technology use is essential. Applications that fulfil these domains are more likely to be chosen over those that don’t. While meeting these preconditions and addressing the need for information can positively influence the choice of an assistive technology, these more “ontological” requirements may not be the sole factors that initiate and then drive the decision-making process.

Gaining a better understanding of the contextual factors that influence whether technology is considered as a solution, and their interaction with individual care needs, could promote a more person-centred care approach. In the same light, there might have previously been “(…) an insufficient focus on (necessities) with regard to the development and implementation of assistive technologies.” [9]. Often, the design and development of these technologies prioritize technical feasibility rather than addressing what those affected consider “necessary”.*.

We conducted a qualitative study with people with mild dementia living at home and their support network to explore how contextual factors and necessities influence the decision-making process that might potentially lead to the adoption of assistive technologies to fulfil support and care needs.

* Note that “context” in this respect is not specifically defined a priori; we see it to resonate with a broader array of preferences, requirements, or circumstances intrinsic to the affected individuals and their network, going beyond technical requirements and immediate care needs. Furthermore, we conceptually distinguish between care needs resulting from cognitive disabilities or health issues and “necessities” that the technology should fulfil. While it is essential for a technology to address a specific care need, additional factors may be necessary for its adoption as a support option, even if they are not directly linked to the underlying care need.

## Methods

### Design, recruitment and participants

A two-phase sequential study was conducted to address the research question. The first phase involved individual interviews (henceforth the “interviews”) with people with dementia (PWD) and their support and/or care-network (predominantly their respective partners). The second phase comprised focus groups with PWD, relatives, and companions (“companions” means friends or close acquaintances).

Recruitment aimed to include a heterogeneous group of individuals with dementia living at home, along with their relatives and companions. A community-based campaign was conducted, involving various care providers and services in Bielefeld, Germany. Local newspaper advertisements were also used to reach individuals who might not have been in contact with formalized care yet. The response rate ensured a sample size and composition that achieved theoretical saturation (although see the Discussion for limitations) [10].

8 PWD (3 female, 5 male; age: mean 79.5, interquartile range 75–80) and 7 partners (6 female, 1 male; age: mean 80, interquartile range 75–86) were recruited for the interviews. These relatives were involved in some form of informal care, albeit only basic support.

We conducted 7 separate focus groups with a total of 29 participants (minimum group size 3, maximum 7). 8 participants (4 female, 4 male; age: mean 78, interquartile range 75–79) were people with dementia. 21 participants were relatives or companions (14 female, 7 male; age: mean 75, interquartile range 67–78); relatives made up about half of the latter group, almost all being the partners of affected. One of the focus groups was comprised of 6 participants of the prior interviews (3 PWD, 3 partners). 

Therefore, information is from 38 individuals in total.

Half the participants had completed high-school or received a higher diploma, the others a maximum 10-year school education.

Dementia was diagnosed by an outpatient specialist (either a neurologist or an old age psychiatrist) independently of and prior to the study. We integrated orientation and language categories of the German translation of the “Mini-mental state examination” into the recruitment interview. The intention was to ensure that people with dementia were able to take informed decisions (Note that none of the PWD individuals applying to take part in the study had to be excluded post recruitment interview.). The (advertisement and subsequent) recruitment strategy resulted in the fact that only individuals with mild dementia were included.

None of the participants had previous experience with assistive technologies designed for dementia-related impairments.

### Study setting

#### Interviews

The interviews were conducted in the homes of the participants (by SP, TG; author initials). The individuals with dementia had the option to be interviewed alone or with their respective relative; all but one chose to have their partner present. The interviews took place between October 2018 and May 2019 and lasted 30 to 60 min. Prior to the interviews, there was a recruitment contact to address procedural questions.

#### Focus groups

The focus groups took place in a neutral meeting room with participants seated freely around a U-shaped table. A moderator and co-moderator (SP, TG sat at the head of the table, with the co-moderator responsible for visible documentation on a pin board. The focus groups consisted of two phases. In the first phase, fictional characters illustrating support needs were introduced verbally, followed by an open discussion. The second phase involved the presentation of “symbols” of technology (e.g. tablets, smartphones, robots; as cardboard depictions) to stimulate focused communication. The moderator maintained the main focus while encouraging participants to respond to each other’s ideas. The focus groups took place between July 2019 and October 2020 and lasted 40 to 70 min [11–13]. 

### Qualitative approach and research paradigm

We started with a formalized construction of the interview guidelines [14], considering relevant literature including a review on technology-assisted home care for people with dementia and their relatives [4], and the expertise of the research group members hailing from various disciplines and professions (nursing and medical professionals, specializing in geriatrics, neurology and geriatric psychiatry (SP, TG, AN, ET, SHK), public health sciences (AN, ET, LS, CD), sociology (GE, CK) and gerontology (TG)); (see the additional file for its abbreviated, translated version). The interview guidelines were constructed specifically for this study. Problem-centred interview methodology was chosen, aiming to capture topic-specific actions, interactions, and subjective perceptions impartially [15, 16]. The analysis of interviews followed the principles of Grounded Theory [17]– producing preliminary conceptual categories. The guidelines for the focus group were motivated by these results. For the analysis of focus groups, Kuckartz’s Structuring Content Analysis was employed to portray heterogeneity efficiently [18]– working with the prior category set (expanding, pruning), finalizing with main thematic categories. This framework was developed iteratively, resulting in a preliminary model [19].

We employed an open, inductive analysis and introduced sensitization concepts derived from previous knowledge [4, 20]. These concepts addressed basic care needs, experiences of coping with dementia, changes due to digitalization, contextual factors, potential necessities that technology should fulfil, general [21, 22], and specific needs theories [23]. Memos were created during the analysis to document insights, open codes, and decisions. Reflexivity was maintained through discussions within the research team, challenging assumptions and addressing dense passages. Two members of the research team conducted the coding (SP, TG). Data were carefully reviewed for any deviant cases that did not fit the categories as a test of “goodness-of-fit”. Digital recordings of interviews and focus groups were transcribed verbatim. Field notes were taken before and after data collection to provide an overall understanding of participants and the data. MAXQDA software facilitated data analysis, management, and memo creation. 

Informed written consent was given prior to study inclusion. To address the potential limitations of “normal” informed consent practice in light of the group of participants affected by dementia (albeit the fact that they were only mildly impaired), we constructed the consent forms to be limited to one page, using accessible language. Furthermore, the researchers read aloud the content sentence-wise and opened each sentence up for discussion. Ethics approval was obtained (ethics committee of the Universität Bielefeld (application number 2018 − 179)).

## Results

Our qualitative analysis produced the following six main thematic categories. The underlying motive intersecting these is a strong desire to maintain self-determination and independence while minimizing stress and negative emotions. Technology should be “meaningful” on different levels.

- Assessment of (one’s own) existing resources;
- assessment of (potential) adverse effects;
- evaluating the (potential) benefits of assistive technologies;
- (pre-existing) trust in assistive technologies;
- (pre-existing) openness towards assistive technologies;
- information needs.

**Fig. 1:**
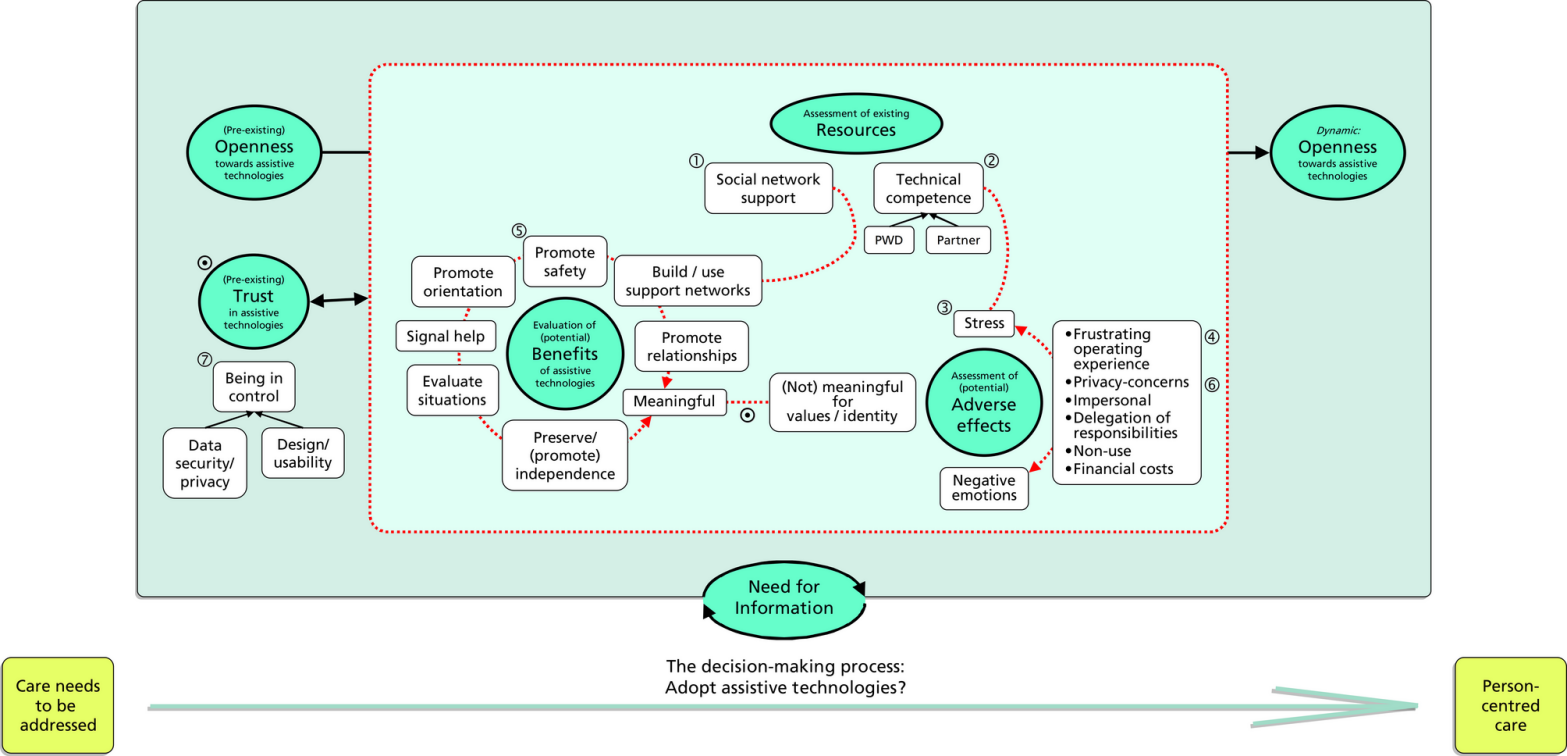
The decision-making process: Will assistive technologies be adopted? The decision-making process would optimally allow for care needs to be addressed in such a way that person-centred care is the result. The aquamarine filled ovals/circles with the thicker outline represent the main thematic categories, while the unfilled boxes in their vicinity denote specific aspects associated with these categories (as explained further in the text). The red-dotted rectangle and lines/arrows signal a grouping or an implicit (or explicit) association between these. Black arrows highlight a more actionable (or temporal) relationship or input. The “assessment of (one’s own) existing resources”, the “assessment of (potential) adverse effects” (promoting stress and negative emotions) and the “evaluation of the (potential) benefits of assistive technologies” can be seen to primarily articulate contextual factors relevant to the decision-making; these common semantics are signalled by placing these main categories together within the red-dotted rectangle. For example, in this model, given that there is a resilient ① social support network, perhaps abating not quite fully sufficient ② personal technical competence, firewalling ③ potential stress– resulting from ④ a frustrating operating experience–, by e.g. taking care of the set-up process of a digital application, these circumstances (i.e. the “context”) may foster choice in favour of assistive technologies. “Benefits” of a technology (e.g. promoting safety, orientation, relationships, etc.) need, however, be apparent - and these are closely related to its technical feasibility, and are clustered correspondingly. They attribute practical meaningfulness, by e.g. ⑤ promoting safety; on a meta-level, meaningfulness– rather the lack of– can be triggered by a perceived disruption of values or identity. Meaningfulness ⁄ and trust ⁄ are necessities that might counterbalance contextual deficits. Again, as an example, if a safety device is associated with ⑥ privacy-concerns in conflict with a personal definition of identity, addressing a ⑦ “feeling of (nonetheless) being in control” might augment trust and promote the use of that assistive technology. Decision-making is influenced by the dynamic aspect of “openness” towards assistive technologies, which can be modified by exposure to, especially, contextual factors. There is a need for information that spans across all stages of the decision-making process and is crucial for informed choices

See the structuring of the possible relationships between the categories in Fig. 1.

Reporting the results, we use a reference to “PWD” or “relatives” (including companions) when the text analysis revealed a specific attribution to either group. More commonly, when neither group specifically initiated content (and its aggregation, as we report it) we use “participants” or leave the ascription blank. Note, however, that one substantial finding was the fact that many aspects of the decision-making process could in fact not be disentangled in respect to who the main contributor was; both the PWD and relatives (and/or companions) were often involved in self-reinforcing narratives.

### Assessment of (one’s own) existing resources

In the decision-making process that might lead to integrating assistive technologies into care routines, participants reported that it will be crucial to reflect on and define the quality and quantity of available resources for the technology’s potential use. The main category “assessment of (one’s own) existing resources” is moderated by two aspects: the potential *support from the social environment* and an *evaluation of technical competence*.

Concerning the *support provided by the social environment*, individuals with dementia (PWD) and their relatives mentioned having previously received assistance for common technology setup and usage (e.g. television sets, phones), particularly from children, grandchildren, or younger neighbours. The perception of a supportive social environment will increase the likelihood of considering the use of specific assistive technology in the future. As cognitive deficits progressed (and will progress), PWD increasingly transferred responsibility for existing technology to their relatives, especially their partners. Consequently, partners anticipated an additional burden of supervising coming assistive technologies.

*“It is so unsettling. I have to look after myself (…). Then I have to take care of the household. Then I’ve got appointments. I have to think for two people*,* organize for two*,* everything. (…) And then I turn on the technology and see what the robot’s doing. Well*,* I think that would be too much for me.” (Relative*,* FG3*,* 65)*.

Some relatives, especially those with limited technical experience or where the cognitively impaired partner previously managed technical appliances, expected difficulties integrating assistive technology into their daily lives. These relatives viewed it as a potential added stressor and expressed a need for support in setup, troubleshooting, and testing in everyday situations.

The *assessment of participants’ own technical competence* depends on their understanding and proficiency in handling technology, and is strongly influenced by their self-image. Some relatives found it challenging to evaluate the PWD’s technical competence (and how long it would be retained due to the progressive nature of dementia) and often rated it lower than the affected person’s self-assessment.

Some participants expressed trust in their own learning abilities and tackled challenges with a constructive mindset. Regardless of others’ opinions, they considered themselves more competent (and with more resources) in handling technology. This was particularly relevant when these assessments differed between the parties.

*“I would never say I can’t do this*,* I can’t manage that*,* I would always try it again and again.” (PWD*,* EB08*,* 214)*.

On the other hand, some participants perceived themselves as technically inexperienced and lacked confidence in their abilities to understand and operate technologies correctly. Their limited self-competence was associated with a greater desire to conserve their resources and highlighted their dependence on the immediate social environment.

### Assessment of (potential) adverse effects

The participants underlined a tension between the expected adverse effects of using assistive technology and a potential detriment if they (actively) decided not to use it.

Avoiding negative emotions such as anxiety or insecurity was a significant motive. The term “adverse” referred to anything that might cause stress, particularly from the perspective of those with cognitive impairments.

*“Well*,* I think (I) would get scared too*,* if suddenly a voice came from the clock saying ’Everything is alright’”. (PWD*,* FG6*,* 61)*

Individuals with dementia may perceive certain assistive technologies as challenging or frightening to operate, leading to frustration and feelings of failure. Consequently, these participants expressed negative attitudes toward technology.

*“But that’s difficult because I forget certain processes very quickly*,* I’d have to*,* well I’d have to do it all the time to keep it in my mind and be able to bring it up when I need it (…).” (PWD*,* EB03*,* 92)*.

Another aspect of adversity was the feeling of being surveilled by technology. For example, the need for location monitoring for safety reasons was questioned, considering the potential invasion of privacy; participants often reflected the perspective of their children and adopted their suggestions in this respect.

Further negative effects attributed to assistive technologies such as robotics, monitoring systems or telehealth applications were that they might result in less personal and physical attention, direct communication and social interaction.

*“And I don’t think technology should replace people either. So you don’t tell someone concerned*,* I’ll give you the best technology and leave you alone with it.” (Relative*,* FG4*,* 93)*.

Discussions also focused on the ethical considerations of adopting assistive technologies and the responsibility of society as a whole for providing care. Participants expressed concerns about leaving individuals with dementia alone with technology and viewed it as a potential substitute for supplementary care.

Given that individual risk exists irrespective of cognitive impairment, it was, at times, questioned, if assistive technologies could “really” contribute to minimizing these risks.

*“For the very simple reason*,* when people run away or do something or somehow have an accident*,* then it just happens. But I don’t see that as too bad*,* because it’s normal and it happens to every person. Most deaths (in people without dementia) happen in the household*,* you can say.” (Relative*,* FG6*,* 34)*.

Participants also reflected on the costs of technologies. They differentiated that there will be costs involved in their acquisition, but also that costs may accrue if they chose not to use then. An example would be that one’s own home could be refurbished to include necessary assistive technologies (at a cost). Refraining from doing so might, on the other hand, prevent them from continuing to live at home because certain care requirements would then not be met. Relocation would also be costly. They advocated for reimbursement of necessary assistive technologies by health or care insurance providers to avoid social selection based on costs.

*“Then it shouldn’t be a technology for those with high income or people with good pensions (only)*,* but that it is also paid for by the health insurance companies. Otherwise*,* I think it would be a new way into a three-class society.” (PWD*,* FG4*,* 133)*.

A recurring theme was adversity that would be induced if technologies were perceived not to have value. “Value” here suggests that technology should transport security, comfort and not disrupt identity, in short: it should be perceived to be meaningful.

### Evaluating the (potential) benefits of assistive technologies

This main category encompasses various thematic aspects related to the attribution of potential benefit to assistive technologies, focusing on their usefulness (being able to solve current care problems, address unmet needs) and practical, technical preconditions– leading to, in a more practical sense, the preservation of self-determination.

*“My basic attitude is*,* (…) if there is something that is useful*,* then that’s what I need.” (PWD*,* EB01*,* 127)*.

The participants emphasized the need for assistive technologies to be useful and thereby surpass analogue solutions. Concerns were raised about the additional effort required to adapt to technology. Usefulness was also evaluated based on integrability into everyday life and existing care structures.

This practical “meaningfulness” and resource orientation were crucial for participants when selecting suitable interventions to structure their care situations.

A dominant theme emerged from the analysis, highlighting the participants’ desire to *preserve (and promote) (physical) independence*. Technologies would be considered useful if they enabled unconstrained activity and allowed for periods of absence by relatives. For example, a tracking system was discussed to facilitate being outdoors for individuals with dementia and orientation difficulties.

*“(…) where I think independence is still maintained a little. The person can go for a walk without being so afraid of getting lost or simply that you know where he is a quarter of an hour later or half an hour.” (Relative*,* FG2*,* 129 ff.)*

Assistive technologies would also be valued for potentially supporting everyday activities like cooking, shopping, hobbies, and encouraging routine activities. The hope was that these technologies could positively compensate functional deficits due to the progression of the illness.

The interviews and focus groups revealed diverse needs, leading to the exploration of specific technologies best suited to address those needs.

The theme of *promoting orientation* encompassed technologies that would aid in temporal, situational, and local orientation. Technologies providing time and date information, or supporting day-night routines through lighting systems were seen as beneficial for temporal orientation. E.g. relatives expressed hope that specific light fixtures could automatically illuminate rooms, supporting spatial orientation and reducing the risk of falls. Moreover, situational orientation could be enhanced by systems that automatically notify of, for example, doctors’ appointments, inform relatives and facilitate communication with the person with dementia.

*“That the necessary information is sent to the patient in advance. (…) So you could talk about it before at home. And say: ‘This is how it will be.’ Thus*,* for the patient*,* avoid frustration of not understanding.“ (Relative*,* FG1*,* 74)*.

Assistive technologies enabling better understanding of the PWD’s situation, and *assessing situations* and the need for action were viewed positively by the participants, particularly in cases where he or she lived alone and relatives needed to contact them promptly for reassurance.

*“To be able to see*,* listen and say something from afar. (…) (S)ometimes it happens at night and there’s been a phone call: ’I’m scared*,* someone was here.’ Probably a dream*,* so you drive over. But that doesn’t help at that moment either. Because by then he or she has (…) already forgotten it.” (Relative*,* FG6*,* 93)*.

There is a desire to be able to *indicate when help is needed*. Technologies that could provide location or health data and can be shared with healthcare providers or relatives were considered to be potentially useful. Appliances that record changes in a situation based on vital signs or behaviour (especially if rapid medical assessment became necessary) are seen as helpful, as the need for help is dynamic and unpredictable.

*“Or that you can have an agreement with your GP or attending physicians. If so-and-so gets on the website*,* the medical assistant there automatically calls the patient. This means that the affected person doesn’t have to dial any numbers and doesn’t get confused. It’s the medical assistant who takes the initiative.” (Relative*,* FG1*,* 48)*.

The advantage of technologies acting independently of human input was seen to be of additional benefit.

Technologies could also *promote safety* by reducing the PWD’s fear of harm and relatives concern. Medication management systems and equipment safety measures were mentioned.

*“You would have to have compartments for the various medications that are then released at a certain time and a signal comes. And if you haven’t taken it*,* a signal must also come*,* somehow.” (Relative*,* FG2*,* 91)*.

Telehealth or reminder systems were expected to reduce uncertainty.

*Building (or maintaining) and using support networks* was seen to be essential for future independence. Technologies enabling digital communication were valued. Participants highlighted the need for such networks supporting household tasks.

*“And that it takes on heavier tasks in the house. (…) Yes*,* for example*,* helping the partner doing the caring at home.” (Relative*,* FG2*,* 102ff.)*

Participants expressed a positive response to technologies that might *promote personal relationships*. Specifically, technologies enabling a “tele-presence”, such as video systems, were praised. The participants emphasized their desire for security, independence, and stronger connections with others, indicating that technologies addressing these needs could be beneficial.

### (Pre-existing) trust in assistive technologies

A crucial requirement for assistive technologies is to instill a sense of “trust” and “being in control.” This is influenced by rather disparate factors such as data privacy and usability issues (design, adaptability to individual characteristics, etc.).

Specifically, participants emphasized the importance of clear information on data handling to reduce anxiety and enhance trust in the technology.

*“And also with regard to data security*,* I really think I have to say honestly I find this incredibly important. Where does the data go to?” (Relative*,* FG4*,* 130)*.

The participants expressed confidence in assistive technologies that “worked well”. They also emphasized the importance of human-technology interaction, particularly when the technology was flexible and adaptable to individual characteristics and current needs, such as communication abilities.

Usability played a crucial role in building trust, as participants found difficulties in using technology to be trust-deterrent. They preferred a minimalist, intuitive and consistent user experience that could be used by individuals with dementia over an extended period.

It was agreed that technologies should have a clearly defined purpose and offer only a few adaptable features aligned with user habits. Recognisability was also important, with familiar design patterns and a pleasant appearance, potentially resembling humans or animals, helping users establish a quicker connection with the technology.

*“I think the (robot) seal is quite nice*,* it’s a*,* well*,* it has something safe about it. Yes*,* a cuddly toy*,* that’s lovely. (…) Because a cuddly toy means something safe for me*,* like from the past.” (Relative*,* FG6*,* 79)*.

### (Pre-existing) openness towards assistive technologies

Openness towards the future use of technology is influenced by self-perceived technical competence and anticipated needs that could be fulfilled by assistive technologies. It is also shaped by one’s understanding of the illness, which may lead to a complete rejection of technology if the mindset is that people with dementia are unable to use it due to cognitive impairment.

Openness reflected the participants’ willingness to consider assistive technologies at all as a potential solution for care problems, based on their personal understanding of the illness. It involves considering technology-based solutions in general or for specific use-cases. It’s worth noting that comments related to openness were often expressed early on in interviews or focus group sessions, either directly or in response to specific technology examples.

*“To go for a walk by the lake and not end up in (some place)*,* but (having) to find your way back home. I mean*,* if it were necessary*,* of course I would*,* yes. But (…) a wheelchair with GPS navigation (laughs). Yes. That would be something. (…) Yes*,* well*,* I am open to that.” (PWD*,* EB01*,* 81)*.

The initial degree of openness to technology was not fixed and could change through discussion, exposure to technology and context. It was found to be a dynamic variable of the decision-making process.

### Information needs

A recurring theme in the interviews and focus groups was the strong desire for comprehensive information. This encompassed details about assistive technologies, operating instructions, initial setup, contractual conditions, and the option for obligation-free testing to assess integration with existing needs and lifestyle.

*“I’d downloaded an app for this memory training*,* (…) seven trial days free of charge and then the subscription would cost*,* I don’t know what. Now*,* I’m quite honest*,* you could use this trial phase first*,* but how do I get out of it again without the contract coming into force after the trial period.” (PWD*,* EB06*,* 164)*.

Participants commonly relied on their social environment as a source of information. The perceived benefits and usability played a significant role in their motivation to seek further information.

We opted against a study design that would have allowed for an investigation of information needs in respect to specific (or groups of) technologies (e.g. design of manuals, concrete information on benefits or detriments, etc.). Rather content that was aggregated to this thematic category intersected other topics fluidly and cropped up freely in the discussions (e.g. above quote). Reverberating previous mentions, participants highlighted following aspects:

Information provided on technologies

- should be individualized, reflecting past experience, competence and reference the level of assistance previously received from others;
- should address benefits of its use, with reference to specific needs articulated;
- should highlight and discuss potential adverse effects openly, not least
- to instil trust in the technology and allow users to judge if they would feel in control.
- As with the technology itself, information should be “minimalist, intuitive and [deliver] a consistent user experience”.

## Discussion

The results highlight that the decision-making process that might lead to the adoption of assistive technologies is complex– involving an intricate negotiating process involving the individual with dementia and his or her social environment. It is by no means pre-determined solely by if a technology can adequately technically attend to a specific support or care need. A review of studies investigating facilitators and barriers of assistive technology use goes as far as saying that the care-network “need[s] to be motivated to use [assistive technologies] i.e., understand how they can benefit personally before they are willing to accept […] its use.” [24]. This “motivating” subsumes the process of matching pre-existing expectations with the actual use-case. We come to the same conclusion as others, that the “margin between whether […] specific care interventions [are] perceived as supportive or infringing [is] small […]” [25] and highly individual.

While the goal of preserving self-determination and independence in one’s own home environment through assistive technology is prominent, the perception of potential adverse effects and the lack of sufficient resources can impact the decision-making process; again, both the “risk” of adverse effects and the level of what is deemed “sufficient” in respect to resources is highly individual.

On the other hand, a positive perception can be fostered when specific technologies are seen as meaningful, providing security and practical benefits such as enhanced social support, interaction, and a sense of belonging. The importance of addressing these “values”– as was identified in this study– has recently been underlined elsewhere, and this "addressing" should start at the very begining of the design process [26].

When planning and solving care problems, the selection of interventions that meet individual care needs is influenced by the participants’ pre-existing openness towards assistive technology. This openness is shaped by their initial opinions and their concept of the illness, and it can change during discussions or exposure to technology; i.e. decision-making is a dynamic process pre and also post-implementation [27, 28]. Additionally, there is a strong desire for easily understandable information about specific assistive technologies.

In the Introduction we postulated that a better understanding of contextual factors of the decision-making, interacting with specific care needs, and a focus on “necessities” of those affected and their support network might promote the consideration of assistive technology. While it was not possible to attribute the “semantics” of these prerequisites to any one of the main thematic categories specifically, the categories did reflect and aggregate them differentially. Based on this, we propose a preliminary model illustrating the decision-making process (see Fig. 1). While none of the constituent parts of the model are novel per se [29], we do believe that considering the respective connotations of the main thematic categories and their potential interrelationships might facilitate the identification of characteristics that assistive technologies should have to enhance their development. This understanding may also benefit health and other professions in counselling individuals on their adoption, thereby promoting more person-centred use cases.

The influence of additional factors on the decision-making process, however, remains unclear. These factors could include prioritizing specific care needs over others, the valuation of what necessities the technology should fulfil depending on different strategies of coping with the illness [30], or when there is an acute crises, or, importantly, specific cultural/societal influences.

To address the complexity associated with the decision-making process, it seems crucial to provide comprehensive information and education about assistive technologies in the early stages of the illness. This becomes particularly important as the disease progresses and the support network faces greater stress and limited resources to make immediate care decisions [31].

Based on our findings, important topics to consider in educational interventions include (1) integrating past technology experiences, understanding the values, expectations, and fears of both the person with dementia and their relatives, (2) managing realistic performance expectations through the opportunity to assess different technologies without obligation, and (3) actively developing strategies for integrating assistive technology into everyday life and existing care provision.

Additionally, it is important to acknowledge the challenges that arise when the responsibility of technology use shifts to others (e.g. the partner) as the cognitive disabilities of the person with dementia worsen. Proactive measures should be taken to address these dynamics and potential burdens that may arise.

### Limitations

Our study focused on recruiting a diverse group of individuals with dementia and their support network who were still living at home. Our findings can therefore not be directly extrapolated to other settings such as care homes [32]. In addition, our sample excluded individuals with severer cognitive deficits, so the study did not address specific support options for this group; our model of the decision-making process will not be readily transportable to later stages of dementia.

The sampling strategy allowed for a diverse range of participants. However, it is worth noting that the socio-demographic characteristics of the recruited participants indicate a potential self-selection bias (e.g [33]), with underrepresentation of individuals with lower socioeconomic status or a migration background. Additionally, the recruitment strategy emphasizing “assistive technologies” likely attracted individuals who were more familiar and comfortable with technology as such.

Most of the participants’ interactions were made in the presence of their respective relatives or companions; this could introduce biases, including socially desirable statements. Contributions by different individuals were not, and often could not be differentiated based on those affected by dementia versus other participants. Interconnected with this limitation is the fact that an analysis of important role-related aspects like gender was not specifically carried out. Contrary to what we had hoped for at the onset of the research process, specifically highlighting differences in contribution to the decision-making process by those affected by dementia versus other members of the care-network would have necessitated e.g. single, isolated interviewing. Although this lack of attribution is at least regrettable, it on the other hand highlights how tightly knit, quasi dyadic/polyadic (in our sample at least) the decision-making process seems to be. It is a product of the care-network itself, which explicitly includes the person with dementia.

It is important to note that our focus was on the decision-making process leading to the potential use of assistive technologies. We did not investigate whether decision-making aspects would have been reported differently by participants who had already adopted assistive technologies designed for dementia-related impairments.

## Conclusion

While assistive technologies have the potential to enhance care and support in people with dementia living at home, their adoption is influenced by factors beyond technical feasibility. The decision to use technological support is driven by the desire to maintain self-determination and independence while avoiding aversion, such as additional stress and loss of control. It is crucial that the technology is perceived as “meaningful” on various levels. To facilitate the decision-making process, addressing these factors, along with an existing openness towards assistive technology and providing comprehensive information tailored to the needs of people with dementia and their support networks, could “tip the scale” towards adoption.

## Electronic supplementary material

Below is the link to the electronic supplementary material.

**Supplementary Material 1**: Translated interview guidelines.

## Data Availability

The interviews (in non-translated, i.e. in German) and accompanying material are available from the corresponding author on reasonable request.

## Abbreviations

PWD: People with dementia
