# Supplementary material for: Adopting assistive technologies for the support and care of people with mild dementia living at home—Understanding the decision-making process: a qualitative study
Source: BMC Geriatr. 2026 Feb 5;26:295. doi: 10.1186/s12877-025-05945-2 (PMC12958600; doi:10.1186/s12877-025-05945-2)
Supplement: Supplementary file 1 — Supplementary Material 1: Translated interview guidelines. [file 12877_2025_5945_MOESM1_ESM.pdf]

Translated interview guidelines

This is a translated (from German) and abbreviated version of the interview guidelines. Note that the questions listed below are prototypes and were not presented literally across all interview settings.

First phase

The first phase is a narrative-generating introduction; the goal being to empathically reflect on the individuals' illness experiences and coping.

Question 1

You mentioned earlier that the first symptoms became noticeable about two months ago. What has changed for you since then?

How have you dealt with these changes?

Question 2

Could you tell me how your daily life has changed since the dementia diagnosis and how you have coped with it?

Second phase

Introduction to the topic and focused questions related to the topic; externally driven but also immanent follow-up questioning on both the previously discussed and novel aspects.

Question 3

You mentioned earlier how you are coping with your changed situation. To what extent do you use technology or technical aids for this?

Question 4

How does technology play a role for you in managing changes brought on by dementia?

Follow-up questions:

- *What specific benefits or advantages does technology have to offer you?*
- *What holds you back from using technological solutions in your current situation?*

Question 5

For technical aids, this could include, for example, emergency or alert systems, household sensor systems, or digital communication systems. Is there anything you are already using in this area?

Question 6

When you think about the different areas of your daily life, in which areas could you imagine technical support in the future?

Areas were introduced ad lib if necessary to support participants answering:

- Household management (bills, shopping, nutrition, repairs, cleaning)

- Mobility (health, physical activity, social participation, errands)
- Personal care
- Medical therapy/doctors' visits (monitoring progress)
- Safety (emergency calls, monitoring)
- Advice and training
- Organization
- Maintaining social networks (social participation)
- Recreation, games, hobbies, creative work, meaningful activities

Furthermore, interview participants were engaged and supported in trying to come up with specific technologies that could be useful in prior settings – without becoming specific at first.

If, however, no specific technologies came to mind, examples are provided, introduced by: "Could you imagine a technology like […] being useful for you?" and then asking for reasons why these technologies could be perceived as being helpful or not.

- Navigation assistants, dementia-friendly clocks, GPS systems
- Apps/websites for cognitive activation
- Household sensor technology:
    - for daily structure and supporting adherence in medication therapy (medication reminders)
    - for capturing and transmitting relevant vital parameters and activity data, or to communicate with care and medical providers (smartwatch, intelligent bed, sensor-based alert system)
- Video-based communication with relatives and care providers
- Apps or websites for information

Question 7

Is there anyone who supports you in using technology, or whom you would like support from?

Follow-up question:

Who would be particularly suited for guiding and helping you implement this?

Question 8

Has your attitude towards technology changed over the years?

Has your feeling or expectations regarding technology shifted recently?

Follow-up question:

Can you illustrate this with an example?

Question 9

If time and money were no issue, what technical support would you wish for in your daily life?

Third phase

Question 10

Is there anything we’ve missed that you feel is important to discuss? Is there anything you would like to add?
